# A New Generation Prothrombin Time Method for INR

**DOI:** 10.2174/1874104500802010011

**Published:** 2008-02-27

**Authors:** Juha Horsti, Helena Uppa, Juhani A Vilpo

**Affiliations:** 1 Helena Uppa, Tampere University Hospital, Centre for Laboratory Medicine, Tampere, Finland; 2 Department of Clinical Chemistry, Centre for Laboratory Medicine, Tampere University Hospital and University of Tampere, Tampere, Finland

**Keywords:** PT, prothrombin time, oral anticoagulant therapy, Pivka.

## Abstract

Prothrombin time (PT) is the leading test for monitoring oral anticoagulation therapy (OAT). We sought to determine INR taking into account only active coagulation factors FII, FVII and FX without inhibition in patient plasmas and calibrator kits.

We measured PT using a combined thromboplastin reagent. The calculation was based on a new PT method, which measures active coagulation factors (F II, F VII, FX) and corrects the errors caused by inactive coagulation factors.

On this basis, an INR result with and without inhibition for individual patient samples was also calculated and applied to 200 plasma samples obtained from OAT patients. Conspicuous variation in inhibition between the four calibration kits was noted. The kinetics of this inhibition was closest to a noncompetitive pattern.

The need of correction for INRs of single patients increases with higher INRs. At the same level of patient INRs the coagulation inhibiton varies markedly.

It has been known that different thromboplastin reagents possess variable sensitivities, but this may depend on sensitivity in inactive coagulation factors. PT methods today measure the sum of active coagulation factors and inhibition of inactive coagulation factors. ISI calibrators contain variable amounts of inactive coagulation factors, which renders harmonisation of INR results.

Application of the Acf-PT (INR_Acf_) presented in this work develops the PT methodology to measure the true coagulation activity *in vivo* for patient warfarin therapy without inhibition. INR_Inh_ can evidently also be used for the diagnostics and follow-up of certain liver diseases.

## INTRODUCTION

Oral anticoagulation therapy (OAT) is - globally - one of the most widely used medications. The benefit of OAT has been proved in many clinical indications, but the major problem with it has been a narrow therapeutic window; overdosing increases the risk of bleeding and less than optimal anticoagulation predisposes to thrombosis. The prothrombin time test (PT) was introduced by Quick more than 70 years ago. It has served as a basis for OAT monitoring from the beginning of this therapy. According to a recent review the problem with this test, however, has been the poor comparability of results obtained in different laboratories [1]. An attempt to solve this problem was by the introduction In the mid-1980s of a universal means of harmonizing and reporting PT results. All thromboplastins should be calibrated in terms of the International Sensitivity Index (ISI) and the PT results given in INR (International Normalized Ratio) units [2]. In practice, standardization should be performed by comparing the results from test thromboplastin with those given by a reference thromboplastin calibrated in accordance with the method recommended by the WHO Expert Committee on Biological Standardization [2-3]. Unfortunately, an increasing body of evidence indicates that this goal has not been achieved using manufactured and local calibrators [4-9].

Many of the circumstances under which PT determination takes place are uncontrollable. These include - at least - that: (i) only a fraction of the blood components involved in the clotting reaction have been characterized [1]; (ii) different reagents are known to vary with respect to source of thromboplastins and other reagent components [10], and (iii); that these have likewise not been characterized in detail.

In the present study we sought to establish whether PT results could be measured by determining PT times taking into account only active coagulation factors FII, FVII and FX, i.e., eliminating the effects of various coagulation inhibitors. These factors lack gamma carboxyglutamic acid, which is necessary for calcium binding and thereby for “adsorption” of these factors to phospholipid surfaces. They are inactive analogues to active coagulation factors [11-13], most of them obviously being PIVKA (Proteins Induced by Vitamin K Absence or Antagonist ). PIVKAs are also circulating markers in hepatocellular carcinoma [14-15] and biliary cirrhosis [16] without warfarin therapy.

## MATERIALS AND METHODOLOGY

### Patients and Blood Sampling

Venous blood samples were obtained from 10 normal subjects and 210 hospital and health-centre patients for whom the PT time test was requested for the monitoring of oral anticoagulant therapy. In our region a “P-INR” test code is used for this purpose. The patient samples thus represented all possible phases of anticoagulation: (i) before treatment, (ii) dose-adjusting phase, and (iii) steady- state phase. All procedures were approved by our institution’s responsible committee in accordance with the Helsinki Declaration of 1975. Blood (1.8 mL) was drawn into citrate coagulation tubes (Greiner Labortechnik GmbH, Vacuette cat. no. 454322, 9NC) containing 0.2 mL 0.109 mol/L (3.2 %) citrate solution. The sample needle (Terumo, Venoject needle, Quick Fit, cat. no. MN-2138MQ) was 0.8 x 40 mm. Sample tubes were centrifuged at 1850 *g* for 10 min at 20 °C to separate plasma. All measurements were commenced within 8 hours of blood collection.

### PT Determination

The PT coagulation times were measured using a fully automated BCS coagulation analyser (DadeBehring Coagulation System, DadeBehring, Marburg, Germany) and Owren PT method (combined thromboplastin reagent). The coagulation reaction contained 10 µL of citrated sample plasma, 60 µL of diluent and 140 µL of reagent for normal PT measurement and 5 µl of citrated sample plasma 65 µL of diluent and 140 µL of reagent for patient and calibrator measurements using the new PT method. Sample volumes were for "linear check": 15 µl, 10 µl, 8 µl, 6µl and 4 µl and dilution factors: 0.67; 1.00; 1.25; 1.67; 2.50.

The test reagent was Nycotest PT, cat. no. 1002488 (rabbit brain thromboplastin) and a diluent (Nycotest PT, dilution liquid, cat.no. 1002485) from Axis-Shield as, lot 10112954, ISI=1.07.

### ISI Calibration

Two local ISI calibrator kits were used: (i) “Svensk nationell kalibrator för protrombinkomplexaktiviet”, from Equalis, lot 11, 12, Cal 1=0.85 INR and Cal 2=3.19 INR (used mainly in Sweden and Norway). (ii) “ISI-kalibraattorikitti”, cat.no. B10000150, from Bioclin, lot 8, Cal 1=2.07 INR, Cal 2=3.52 INR and Cal 3=1.0 INR (used mainly in Finland).

Further, two commercial (“manufacturer calibration”) ISI calibration kits were used: (i) Etaloquick cat. no.00496 from Diagnostica Stago lot 041555. Cal 1=0.91 INR, Cal 2=3.24 INR and Cal 3=4.90 INR. (ii) PT-Multi Calibrator cat.no. OPAT 035 from DadeBehring, lot 35422. Cal 1=1.01 INR, Cal 2=1.30 INR, Cal 3=1.65 INR, Cal 4=2.97 INR, Cal 5=4.00 INR, Cal 6=5.29 INR.

### Determination of Minimal PT Time and Respective INR

The construction of a PT sec (Y-axis) versus C (where C is the dilution factor of normal plasma, OAT plasma, or calibrator) plot shows, at the intercept of the line obtained from the experiment and the X-axis, the so-called minimal clotting time (t_min_) [17]. This latter is the clotting time which would be obtained under the conditions of the test in the presence of an infinite amount of the clotting factor, the inverse concentration of which is rendered on the X-axis. The inhibition effect can be calculated from the differences in intercepts of the unknown sample and normal plasma (or INR “zero” calibrator) (Fig. **1B**). In practice, only two dilutions are required for each determination [17-21].

The inhibition principle on y axis is illustrated in Fig. (**1A**,**B**). We further calculated the difference in intercepts (= inhibition) also in INR units and subtracted it from total INR_Tot._

INR_Acf_ = INR_Tot _– INR_Inh_

INRs were calculated using the formula: INR = ( sample _sec_ / normal _sec _)^ISI^

Patent pending for method (EP 1861720, WO 2006100346).

### Analytical Imprecision and Statistics

The within-run precision of PT tests was measured using one patient plasma sample (n = 10 determinations) with an INR value in the therapeutic range, i.e., approx. 2.2 INR. The respective CV was assessed 1.6 % for Nycotest PT. This is consistent with our previous observations with a broader spectrum of reagents [9]. The Microsoft Excel 5.0 program was used to obtain the correlation functions and INR results.

## RESULTS

The coagulation time versus plasma (calibrator) dilution plots demonstrated a perfect linearity (Fig. **1B**). Furthermore, the convergence pattern of lines demonstrated that the nature of the inhibition was closest to a non-competitive kinetics [20].

Inhibition was demonstrable in all calibrators with INR values greater than 1. As expected, the inhibition increased concomitantly with the increase in calibrator INRs (Table **1**). It is noteworthy, however, that the inhibition varied markedly among the calibrator kits.

**Fig. (2):**
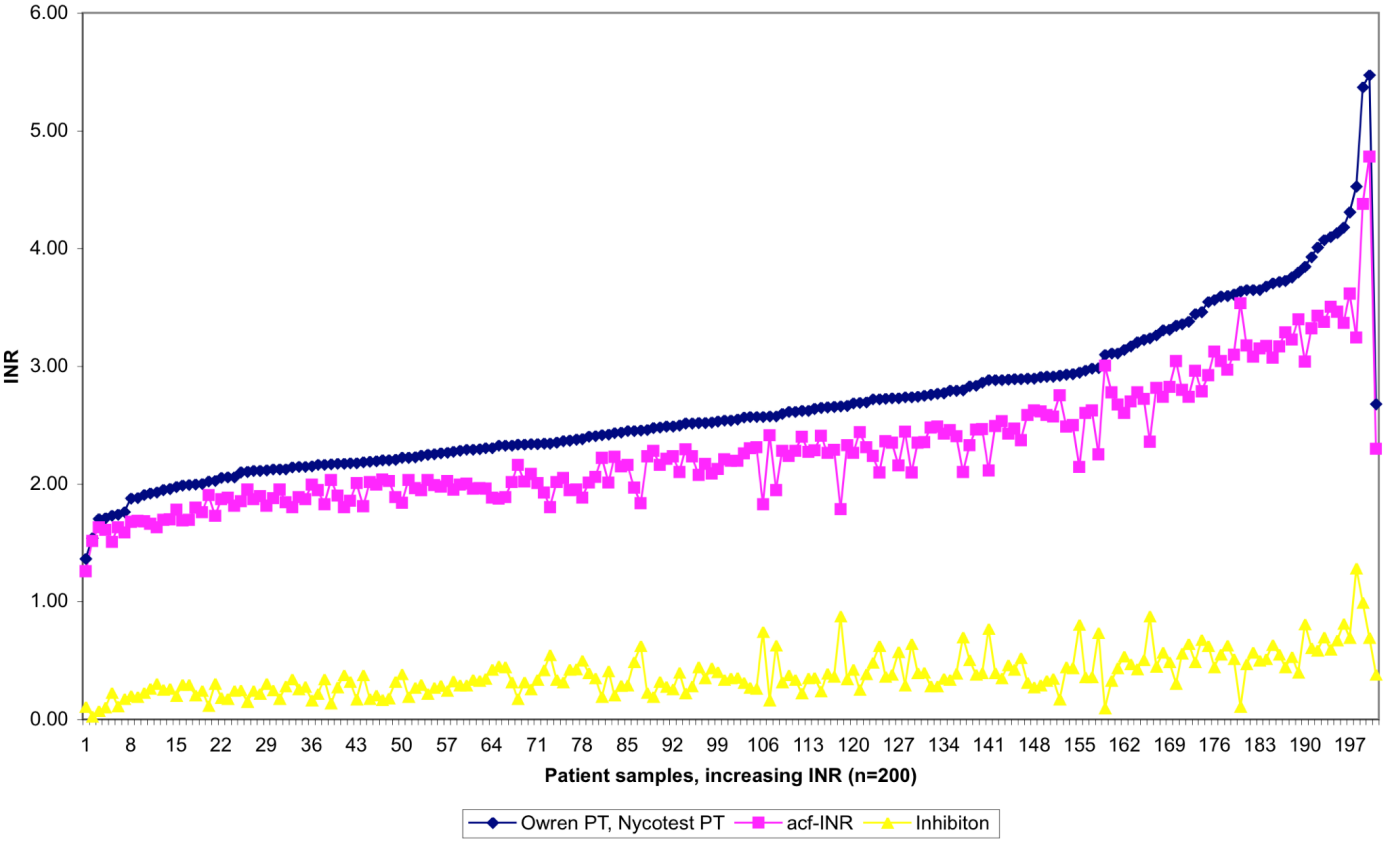
Traditional INR values determined by Owren PT, active coagulation factors (acf-INR) and inhibition effect for 200 OAT patient plasmas in increasing order using Etaloquick calibration.

We further demonstrated that there was a difference - increasing towards higher INR values- between the traditional PT measurement and the new method measuring the active coagulation (INR_Acf_) in the case of the 200 OAT patients. Higher levels of anticoagulant medication decreases the amount of active coagulation factors and inhibition increases proportionally. Individual original and corrected (INR_Acf_) values are illustrated (Fig. **2**). The average for the traditional method was INR 2.68. After correction the average was 2.30 INR units, representing INR_Acf._ The current recommended target point for the OAT is now INR 2.50 (for the most indications 2.0-3.0 INR). The difference between methods is 0.38 INR in other words inhibition at 2.5 INR. These results allowed us to estimate the target point of 2.12 INR_Acf_ units, the range being 1.6-2.6 INR_Acf_ for OAT. The individual variation in coagulation inhibition is notable at different INR levels for OAT patient samples as illustrated in Fig. (**3**) and Table **2**.

## DISCUSSION

Harmonization of PT results and therapeutic ranges globally is an important goal. Equal results from different laboratories would be necessary for the reliable and safe adoption of results obtained by anticoagulation trials and for expert guidelines for treatment. Results from recent studies have unfortunately demonstrated that harmonization attempts have failed badly [1, 9]. Additional confirmation of this unacceptable situation was encountered in this investigation, where a tremendous bias between different calibrators was demonstrated.

The novel approach presented in this paper is based on studies performed by Hemker and co-workers in order to localize, in the coagulation cascade, the site of PIVKA inhibition [19-21]. The authors [19-21] made careful studies and identified factor X analogues as the most important circulating anticoagulants among PIVKA. It is likely that the inhibition effect on factor X also has a central role in OAT plasma- and calibrator-induced inhibition of PTs. At the time in question the PT results were given in seconds or as patient-PT-to-reference-PT ratios. It was precisely the introduction of INR and WHO calibration for INR, which caused the problem of result harmonization for different regents and instruments. The recognition and admission of these inconsistencies is gradually gaining attention. May be this new PT method can help to harmonise INR results [22], because we eliminate the effect of inhibition upon measurement and calibration.

This method requires two measurements and a simple mathematical calculation for one patient sample. It is easy to adapt for different kinds of instruments. The correction is needed for one sample individually and calibrators don’t need to contain coagulation inhibitors (normal plasma dilutions), which helps and develops the global harmonisation of calibration.

In conclusion, today PT methods measure the sum of active coagulation factors and inhibition of inactive coagulation factors. In principle calibrators should not contain inhibitory coagulation factors. The situation is the same using freshly pooled plasmas from normal 20 individuals and 60 OAT patients for calibration according to recommendation [23], because OAT plasmas contain inactive coagulation factors too. Today we have "average inhibition" in PT calibration, which causes disharmonisation using different thromboplastins and reagents.

## CONCLUSION

This new method gives true INRs for active coagulation factors and more accurate care for OAT patients. INR_Inh_ can evidently also be used for the diagnostics and follow-up of certain liver diseases [14-16]. Our hope is that this new approach, based on the INR_Acf_ strategy, would revise the PT method and PT calibration using normal plasma.

## Figures and Tables

**Fig. (1A):**
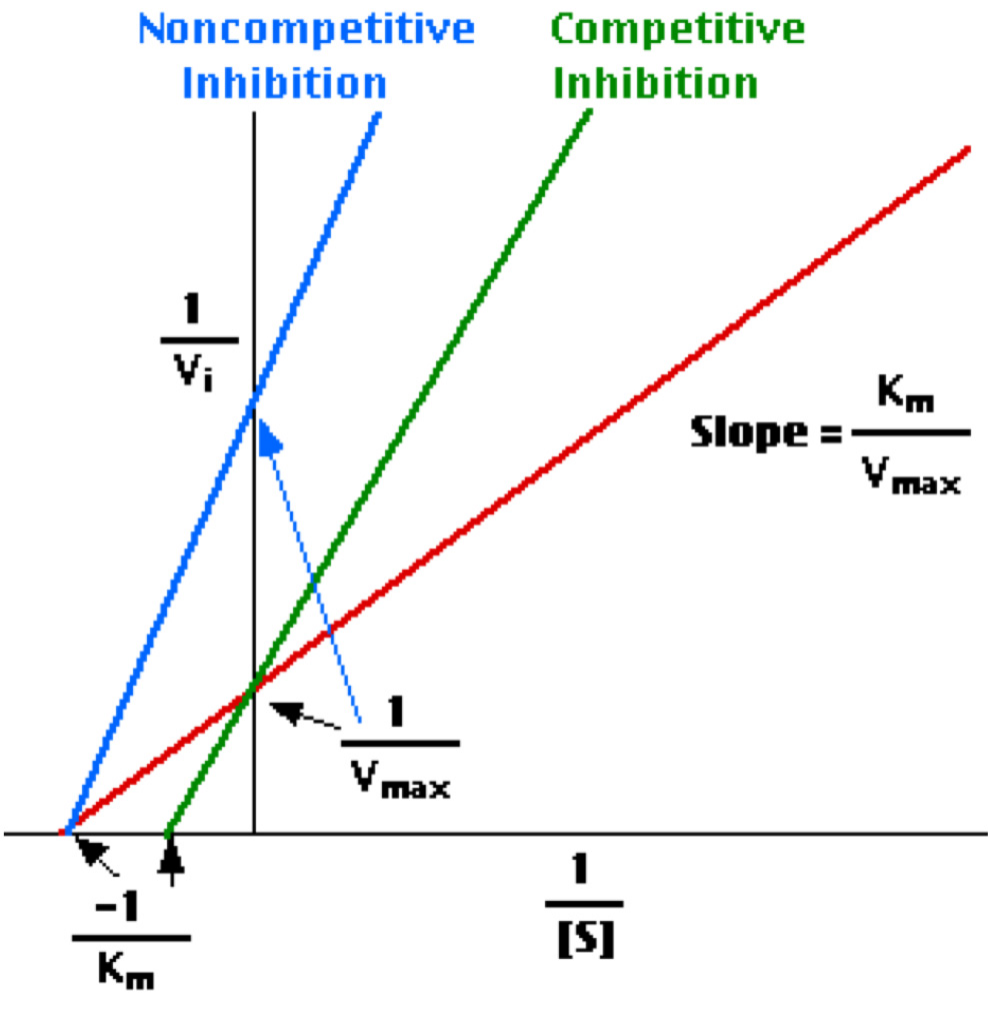
Model of the calculation of inhibition effect.

**Fig. (1B):**
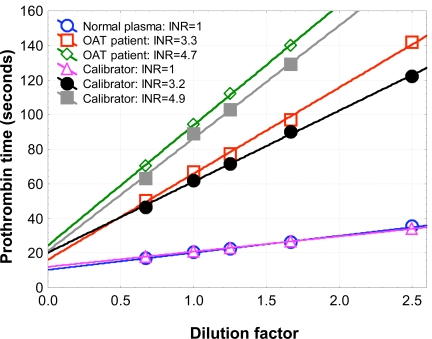
Model of intercept differences between patient plasma and normal plasma on y axis. The difference means inhibition as seconds and INR.

**Fig. (3):**
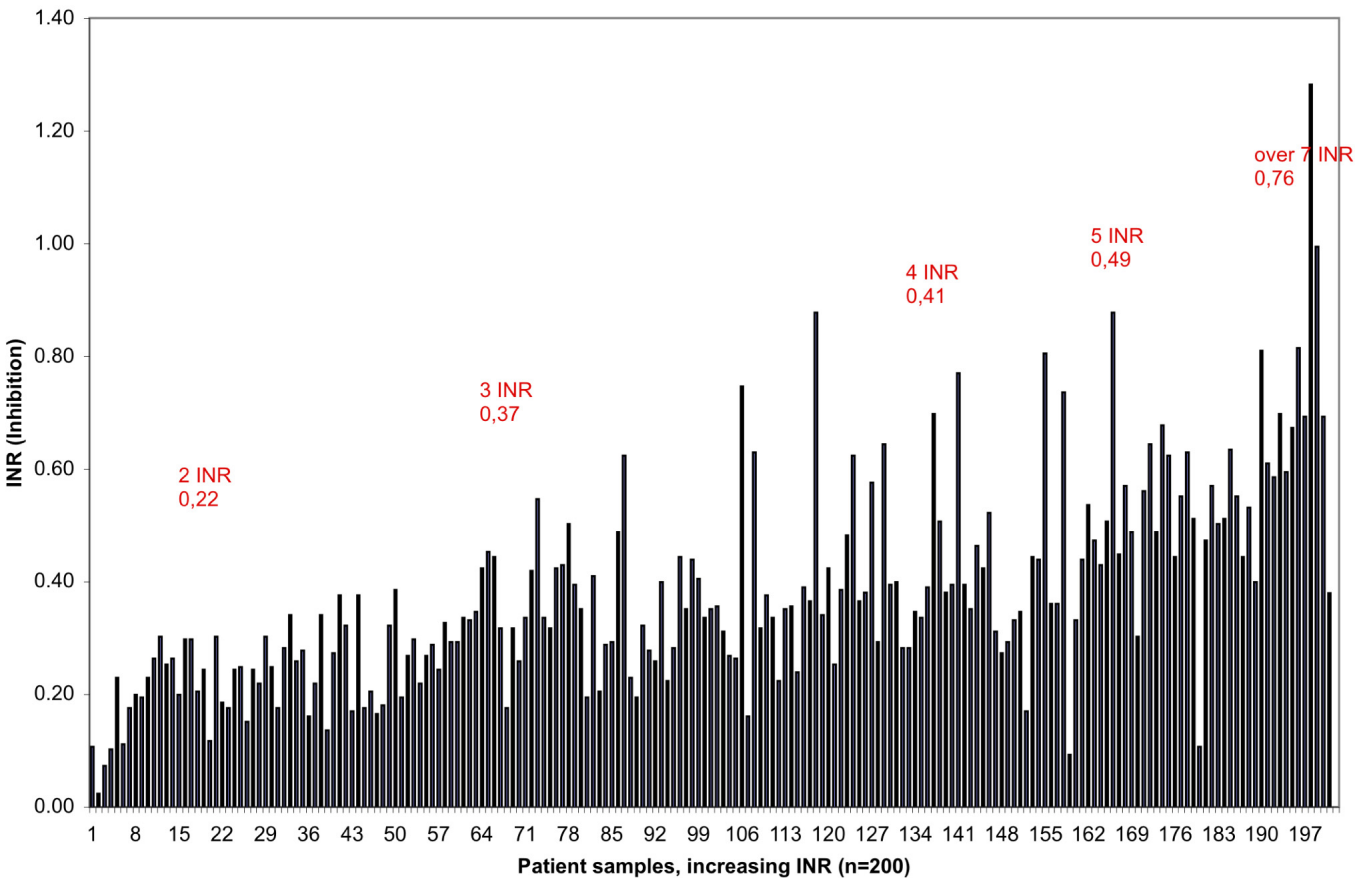
Effect of inhibition (inhibitory factors FII, FVII and FX) on PTs in 200 OAT patient samples in increasing order.

**Table 1:** INR Inhibition Obtained with Four Calibrator Kits Using the Owren PT Method (A Combined Thromboplastin Reagent)

Calibrator^a^	Calibrator INR^b^	INR Inhibition^c^	Active INR^d^	Inhibition (%)
Multical 1	1.01	0.00	1.01	None
Multical 2	1.30	0.04	1.26	3.29
Multical 3	1.66	0.11	1.55	6.76
Multical 4	2.93	0.29	2.64	9.74
Multical 5	4.14	0.49	3.65	11.90
Multical 6	5.46	1.12	4.34	20.43
Etaloquick 1	1.00	0.00	1.00	None
Etaloquick 2	2.85	0.31	2.54	10.78
Etaloquick 3	4.25	1.00	3.25	23.59
Bioclin 3	1.00	0.00	1.00	None
Bioclin 1	2.07	0.32	1.75	15.36
Bioclin 2	3.52	0.74	2.78	21.10
Equalis 1	0.85	0.00	0.85	None
Equalis 2	3.19	0.31	2.88	9.69

a Arranged according to increasing INR values.

b INR given by the manufacturer.

c The inhibition was calculated by subtracting, in turn, the y-axis intercept (INR) value of each respective calibrator of the normal INR value (INR 1.0) from calibrators with higher values, i.e., containing inhibition. For details, see Materials and Methods and Fig. (**1B**).

d The active coagulation (INR_Act_) part of reaction with no inhibitors present.

**Table 2:** Variation of Inhibition at Different INR Levels for OAT Patient Samples

Inhibition n = 10h	2 IN	3 INR	4 INR	5 INR	6 INR	Over 7 INR
Average	0.22	0.37	0.41	0.49	0.49	0.76
SD	0.05	0.11	0.15	0.21	0.15	0.22
CV %	24.41	29.41	36.09	43.57	30,11	28.71

## ABBREVIATIONS

INR: = International Normalized Ratio
ISI: = International Sensitivity Index
OAT: = Oral anticoagulation therapy
PIVKA: = Proteins Induced by Vitamin K Absence or Antagonist
PT: = Prothrombin time
